# Prurigo pigmentosa clinically and immunologically mimicking autoimmune bullous disease: A case report

**DOI:** 10.3389/fmed.2022.1047870

**Published:** 2022-12-02

**Authors:** Kanako Kita, Ichiro Kurokawa, Daisuke Hayashi, Takashi Hashimoto

**Affiliations:** ^1^Department of Dermatology, Meiwa Hospital, Nishinomiya, Japan; ^2^Department of Dermatology, Osaka Metropolitan University Graduate School of Medicine, Osaka, Japan

**Keywords:** autoantibodies, autoimmune bullous diseases, immunoblotting, prurigo pigmentosa, bullous pemphigoid

## Abstract

A 15-year-old Japanese male noticed brown macules on his back 9 months ago. Initial examination revealed reticulated infiltrative erythema and pigmentation with blisters on the erythema of the back. Histopathology showed blisters with eosinophil infiltration in the epidermis, and direct immunofluorescence showed negative results for immunoglobulin (Ig) G, Ig A, Ig M, and C3 in the epidermal basement membrane zone. Immuno-serological tests revealed the presence of IgG antibodies against BP180, linear IgA disease antigen 1 (LAD-1), and laminin α3. The autoimmune bullous disease was suspected, and prednisolone at a concentration of 20 mg/day (0.3 mg/kg/day) was started. When the prednisolone dose was reduced to 10 mg/day, erythema and blisters recurred. The patient was diagnosed with prurigo pigmentosa based on clinical features and was treated successfully with oral doxycycline hydrochloride hydrate and topical tacrolimus ointment. This is the first case of prurigo pigmentosa with blisters in which autoantibodies to the epidermal basement membrane zone were found, which might be secondary non-pathogenic antibodies.

## Introduction

Prurigo pigmentosa, first reported in 1971 by Nagashima (1), generally presents with a highly pruritic urticarial and erythematous rash with recurrent prurigo-like erythematous papules on the back, chest, and neck that leave a coarse reticulated pigmentation (1, 2). It is more commonly observed in women aged 20–30 years, with a male-to-female ratio of 1:4–6.2. Even though the true etiology of the disease remains unknown, prurigo pigmentosa may be linked to diet-related hyperketonemia, eating disorders, and diabetes mellitus (3), as well as sweating and friction caused by clothing.

In this report, we present a case of prurigo pigmentosa with blisters that required differentiation from autoimmune bullous disease.

## Case report

**Figure 1:**
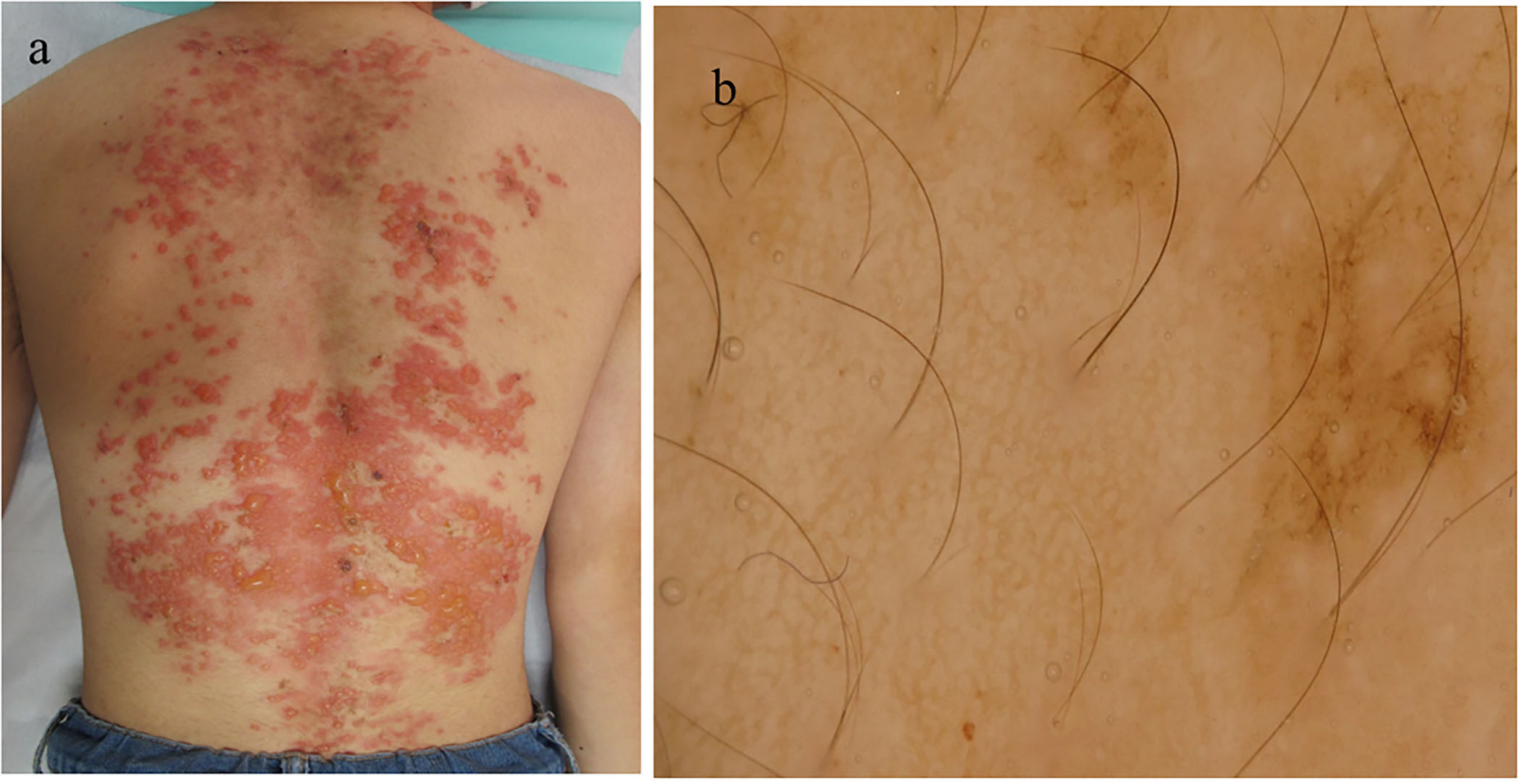
Clinical and histopathological features of the patient during the first visit. **(a)** Clinical features on the back. **(b)** Dermoscopic features.

A 15-year-old Japanese male first developed brownly pigmented macules on the trunk 9 months before his first visit. The skin lesions continued upon topical steroid therapy and then worsened with new blister formation despite treatment with minocycline hydrochloride (100 mg/day). During his first visit, a physical examination revealed extensive exudative erythema and slight pigmentation in a reticulated pattern on the back (Figure 1a). On the erythema, tense and flaccid blisters sized 5–20 mm were observed. His medical and family histories were unremarkable. A dermoscopy of the pigmented area showed a brownish-brown pigment network (Figure 1b). The Darier's sign was negative. The bacterial cultures of the blister content showed positive for *Staphylococcus aureus* (1+).

**Figure 2:**
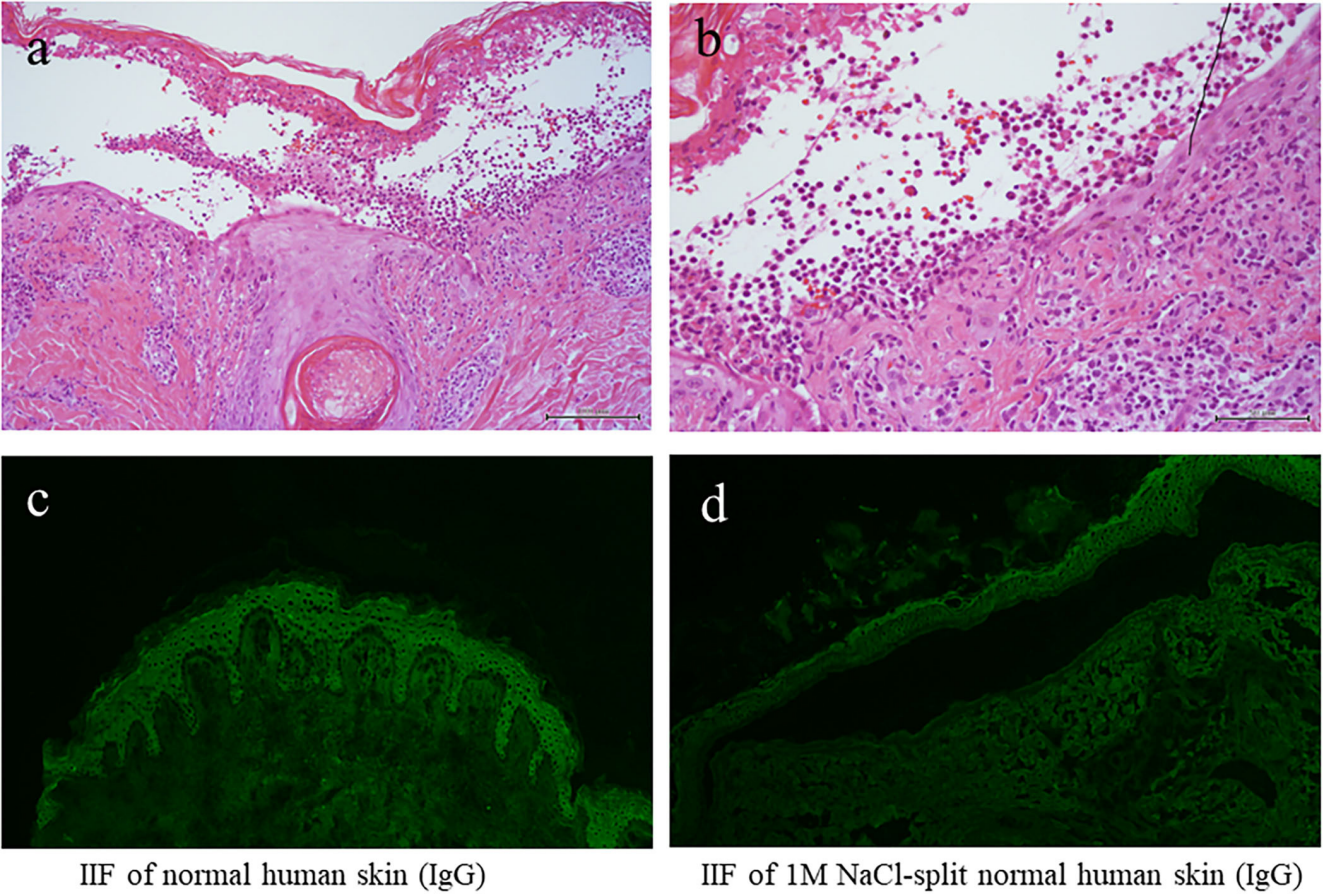
The results of histopathology and IF tests. **(a,b)** Histopathological features [H&E staining; magnifications: **(a)** x100; **(b)** x200]. **(c)** The results of indirect IF of a normal human skin section. **(d)** The results of indirect IF of 1M NaCl split skin section. IF, immunofluorescence.

The results of blood counts and various biochemical tests were unremarkable. The results of anti-nuclear antibodies were negative, and the results of the urinary tests, including urinary ketone bodies, were normal. Histopathology of the biopsy taken from the blistering skin lesion revealed blister formation with eosinophil infiltration in the epidermis (Figures 2a,b). Toluidine blue staining and histochemistry for the c-kit yielded negative results. These histological results, in conjunction with a negative Darier's sign, excluded the diagnosis of mastocytosis and urticaria pigmentosa.

We also performed various diagnostic serological tests for autoimmune bullous diseases, which are routinely performed at our institute (4). Indirect IF of normal human skin sections was negative for both IgG and IgA antibodies (Figure 2c), whereas indirect IF of 1M NaCl-split normal human skin sections showed very weak IgG reactivity on both the epidermal and dermal sides of the split, but no IgA reactivity was detected (Figure 2d).

Enzyme-linked immunosorbent assay (ELISA; MBL, Nagoya, Japan) showed a weak positive reactivity to the BP180 NC16a domain (index 14.5, a cut-off value of <9.0), but was negative for BP230 ELISA (index 0, a cut-off value of <9.0) and type VII collagen (index 2.0, a cut-off value of <6.14). A chemiluminescence enzyme immunoassay (CLEIA; MBL, Nagoya, Japan) for BP180 (5.0 IU, normal 0–8.9) and desmogleins 1 and 3 were all negative.

**Figure 3:**
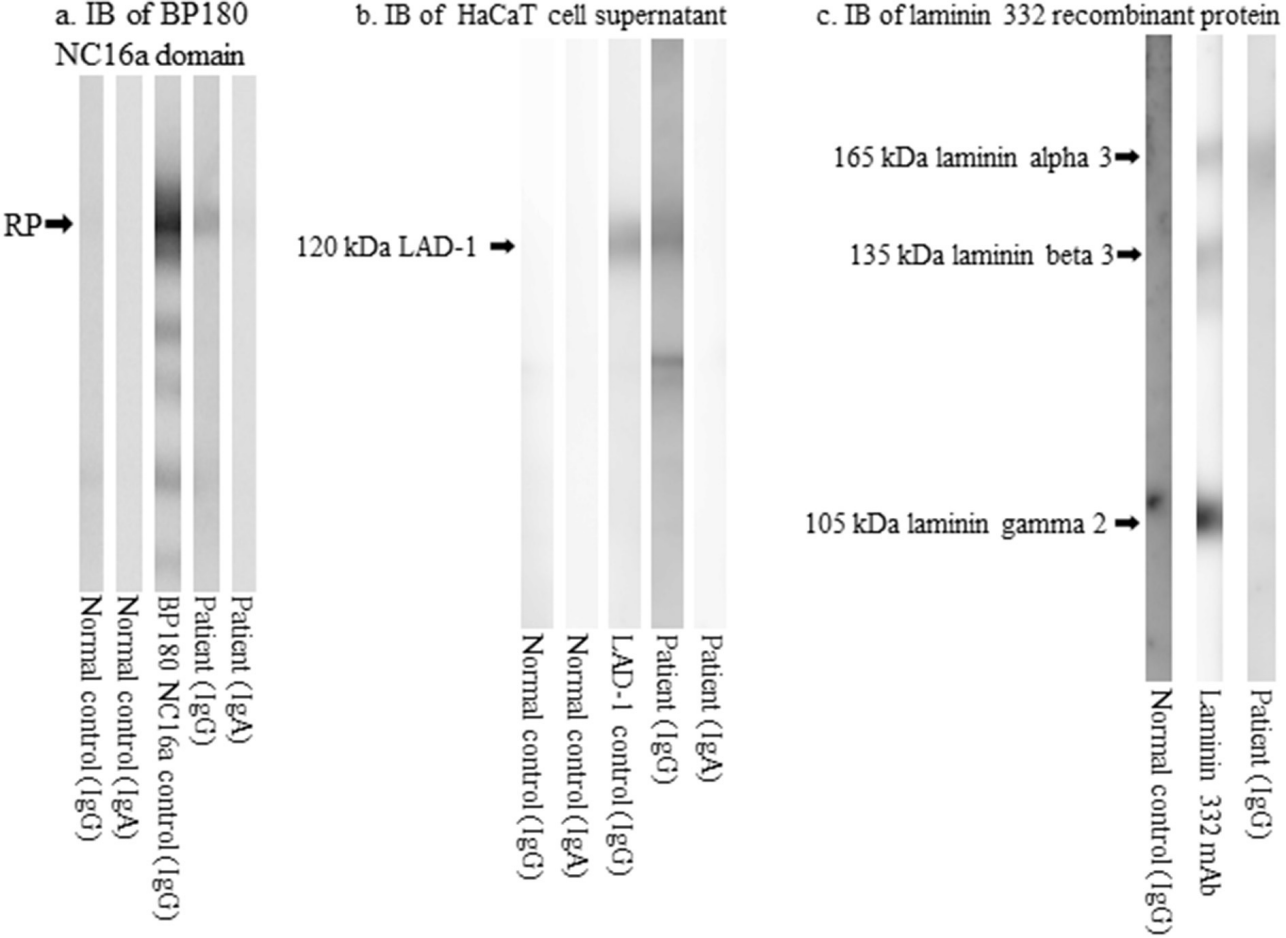
The results of immunoblotting analyses. **(a)** Immunoblotting of the RPs of BP180 NC16a domain showing IgG reactivity with the RPs. **(b)** Immunoblotting of concentrated culture supernatant of HaCaT cells showing IgG reactivity with the 120 kDa LAD-1. **(c)** Immunoblotting of RPs of laminin 332 showing IgG reactivity with the 165 kDa laminin α3. RP, recombinant protein; BP, bullous pemphigoid; LAD-1, linear IgA disease antigen 1; Ig, immunoglobulin.

Immunoblotting analyses showed that IgG, but not IgA, antibodies reacted with the recombinant protein (RP) of the BP180 NC16a domain (Figure 3a); IgG, but not IgA, antibodies reacted with the 120 kDa linear IgA disease antigen 1 (LAD-1) in the concentrated culture supernatant of HaCaT cells (Figure 3b); and IgG antibodies reacted with the 165 kDa laminin α3 subunit of laminin 332 RPs (Figure 3c). Immunoblotting analyses using normal human epidermal and dermal extracts and BP180 C-terminal domain RP showed no positive results for either IgG or IgA antibodies.

**Figure 4:**
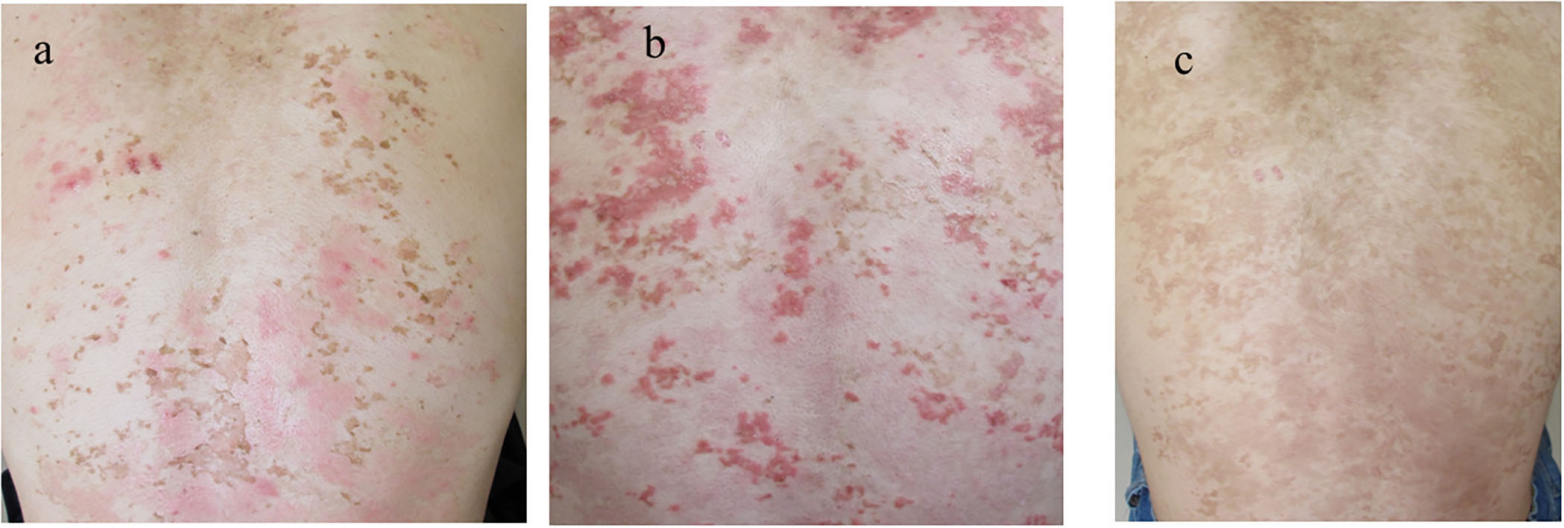
Clinical features during the disease course. **(a)** The skin lesions improved with the first treatment with PIH. **(b)** The recurrence of the skin lesions after the tapering of prednisolone. **(c)** The clearance of erythematous lesions with reticular pigmentation following the second treatment. PIH, post-inflammatory hyperpigmentation.

Upon the suspected diagnosis of autoimmune bullous disease, treatment with oral prednisolone (20 mg/day; 0.3 mg/kg/day), fexofenadine hydrochloride (120 mg/day), and topical betamethasone butyrate propionate was started 9 days after the initial visit. Then, the skin lesions were almost cleared with post-inflammatory hyperpigmentation (PIH) with slight erythema (Figure 4a). When the prednisolone dose was reduced to 10 mg/day, multiple new erythema and blisters recurred on the back with PIH (Figure 4b). Based on the clinical features, a final diagnosis of prurigo pigmentosa was made, and treatment with doxycycline (100 mg/day), oral bepotastine besilate (20 mg/day), and topical tacrolimus ointment was initiated. While the prednisolone was tapered off, erythematous lesions disappeared, leaving reticulated pigmentation (Figure 4c). Thereafter, the patient showed no recurrence.

## Discussion

Although prurigo pigmentosa usually shows no blisters, cases of prurigo pigmentosa with blistering skin lesions have rarely been reported (5–13). Kim et al. reported blister formation in two patients in their cohort of 50 patients with prurigo pigmentosa (5). Hoon et al. reported the case of a 13-year-old male patient with erythema and papules with blistering on the anterior chest and upper back, which were successfully treated with methylprednisolone succinate sodium ester (40 mg/day), doxycycline hydrochloride hydrate (200 mg/day), and tacrolimus ointment for 10 days (6). This case was negative for all anti-BP180 antibodies, antibodies against desmogleins 1 and 3, anti-nuclear antibodies, and anti-DNA antibodies. Yang et al. reported a 20-year-old female patient with pruritic erythematous and blistering skin lesions in a reticulated pattern on the back and neck that was successfully treated with minocycline hydrochloride without recurrence (7). Anti-skin antibodies were not detected. De Francesco et al. (8) and Wang and Xu (9) reported cases of bullous prurigo pigmentosa in which anti-skin antibodies were not detected. Wang and Xu (9), Matsumoto et al. (10), Requena Caballero et al. (11), Kubota et al. (12), and Sun et al. (13) also reported cases with numerous vesicles (12).

Thus, to the best of our knowledge, our patient is the first case of prurigo pigmentosa that developed extensive bullous lesions and showed autoantibodies against the cutaneous BMZ. However, Park et al. (14) reported three cases of non-bullous prurigo pigmentosa with anti-nuclear autoantibodies and suggested the autoimmune nature of prurigo pigmentosa.

Although diphenyl sulfone or dapsone is a therapeutic option for prurigo pigmentosa, minocycline hydrochloride and doxycycline hydrochloride hydrate, which have fewer side effects, have been used in recent years (2, 5, 6). Our patient had not shown improvement with minocycline hydrochloride and hoped to prevent minocycline-induced pigmentation. Therefore, we treated the patient with oral doxycycline hydrochloride hydrate and topical tacrolimus ointment with tapering of oral prednisolone, which quickly cleared the skin lesions, leaving reticulated pigmentation without recurrence. We speculated that the synergistic effects of oral doxycycline and topical tacrolimus would be superior to those of oral minocycline. So, doxycycline hydrochloride hydrate and topical tacrolimus should be good choices for treating prurigo pigmentosa with blister formation.

Since we suspected that our patient had autoimmune bullous diseases, we also performed various diagnostic tests for autoimmune bullous diseases (4), which revealed IgG anti-BMZ antibodies and IgG reactivity with the BP180 NC16a domain, LAD-1, and the laminin α3 subunits of laminin 332. The reason for positive IgG reactivity with recombinant protein of BP180 NC16a domain and LAD-1 but no reactivity in epidermal extracts may be that epitopes on BP180 molecule for IgG antibodies in this patient were exposed in the recombinant protein and LAD-1 but were hidden in the normal human epidermal extract.

Thus, our patient may have had a subepidermal autoimmune bullous disease. However, because the clinical features, particularly reticulated pigmentation, were characteristic of prurigo pigmentosa, the positive results of all the immunological tests were rather weak, and skin lesions in our case were cleared despite the tapering of oral prednisolone. We finally diagnosed our case with prurigo pigmentosa with blisters, and the anti-cutaneous BMZ antibodies were considered to be secondary non-pathogenic antibodies.

However, it is still possible that our patient had both prurigo pigmentosa and autoimmune bullous disease, and that the autoantibodies might be involved in blister formation. In this context, because doxycycline hydrochloride hydrate has recently been shown to be an effective therapeutic option for bullous pemphigoid (BP) (15), it is conceivable that doxycycline and topical tacrolimus also suppressed the autoantibody-causing BP-like blistering lesions in our patient.

Although the definite mechanism for autoantibody production in our patient is currently unclear, autoantibodies may have been produced by exposure to BMZ antigens after damage to the epidermis due to severe inflammation of prurigo pigmentosa. Antibodies against BP antigens were detected in elderly individuals without blistering skin diseases, probably because of the breakdown of immune tolerance due to aging (16). However, this notion cannot be applied because our patient was a 15-year-old.

Indirect IF detected circulating IgG antibodies reactive with the epidermal side of 1M NaCl split skin where BP180 was present. ELISA and immunoblotting showed IgG reactivity with the BP180 NC16a domain and LAD-1 (a truncated form of the C-terminal domain of BP180), confirming the presence of IgG anti-BP180 autoantibodies in our case, which might be involved in the pathogenesis of our case. In contrast, the significance of IgG antibodies to the 165 kDa laminin α3 subunit is obscure because indirect IF did not show IgG reactivity with the dermal side of 1M NaCl-split skin, where laminin 332 is present. Thus, anti-laminin α3 subunit antibodies may not be relevant in our case. Anti-BP180 NC16a antibodies were detected by ELISA but not by CLEIA. This phenomenon is occasionally observed, and we suggest that it is caused by the lower sensitivity of CLEIA than that of ELISA (17).

In conclusion, we presented the case of a boy with rare prurigo pigmentosa with blistering who showed IgG autoantibodies to the epidermal BMZ. Further case series for more cases on this condition are warranted to elucidate the pathogenic role of autoantibodies.

## Data availability statement

The raw data supporting the conclusions of this article will be made available by the authors, without undue reservation.

## Ethics statement

Written informed consent was obtained from the individual for the publication of any potentially identifiable images or data included in this article.

## Author contributions

KK, IK, and TH wrote the original draft and treated the patients accordingly. DH and TH contributed to the immunological methods. All authors have reviewed the manuscript. All authors have contributed to the manuscript and approved the submitted version.

## Conflict of interest

The authors declare that the research was conducted in the absence of any commercial or financial relationships that could be construed as a potential conflict of interest.

## Publisher's note

All claims expressed in this article are solely those of the authors and do not necessarily represent those of their affiliated organizations, or those of the publisher, the editors and the reviewers. Any product that may be evaluated in this article, or claim that may be made by its manufacturer, is not guaranteed or endorsed by the publisher.
